# Supplementary material for: Comparative efficacy and safety of 180 W XPS vs. 120 W HPS GreenLight laser therapy for benign prostatic hyperplasia: a systematic review and meta-analysis
Source: PeerJ. 2024 Nov 27;12:e18615. doi: 10.7717/peerj.18615 (PMC11608016; doi:10.7717/peerj.18615)
Supplement: Supplemental Information 2 [file peerj-12-18615-s002.docx]

**Rationale:**

This systematic review and meta-analysis was undertaken to address a significant gap in the current literature regarding the comparative effectiveness and safety of the 120W HPS and 180W XPS GreenLight laser systems for treating benign prostatic hyperplasia (BPH). Prior studies have evaluated the short-term safety and efficacy of these systems individually; however, comprehensive assessments comparing these two laser systems across clinical outcomes and patient recovery metrics remain sparse. Moreover, with rapid technological advancements enhancing laser system capabilities, an updated evaluation reflecting the latest innovations is crucial. This analysis aims to synthesize existing data to provide evidence-based guidance for clinicians in selecting the most appropriate laser system for BPH treatment, considering operational efficiency and patient safety profiles.

**Contribution to Knowledge:**

This meta-analysis makes a substantive contribution to the field by systematically evaluating and comparing the clinical efficacy and safety of the 120W HPS versus the 180W XPS GreenLight laser systems. By incorporating recent studies and employing rigorous meta-analytical techniques, this work not only corroborates previous findings on operational efficiencies—such as reduced surgery and catheterization times with the 180W system—but also introduces new insights into quality of life improvements and prostate-specific antigen (PSA) level reductions. Significantly, this study highlights differential impacts on minor complications and provides a nuanced view of postoperative outcomes, which is a novel addition to the literature. The findings elucidate the superior performance of the 180W system in managing larger prostate volumes, thereby guiding clinical decision-making. Moreover, this analysis addresses a critical need for updated comparative data that reflects the latest technological advancements in laser treatments for BPH, offering a robust foundation for future clinical guidelines and research initiatives. This work thereby not only fills an existing knowledge gap but also sets a precedent for subsequent studies to explore long-term outcomes and refine BPH treatment protocols using advanced laser systems.
