# Supplementary material for: Comparative efficacy and safety of 180 W XPS vs. 120 W HPS GreenLight laser therapy for benign prostatic hyperplasia: a systematic review and meta-analysis
Source: PeerJ. 2024 Nov 27;12:e18615. doi: 10.7717/peerj.18615 (PMC11608016; doi:10.7717/peerj.18615)
Supplement: Supplemental Information 3 [file peerj-12-18615-s003.docx]

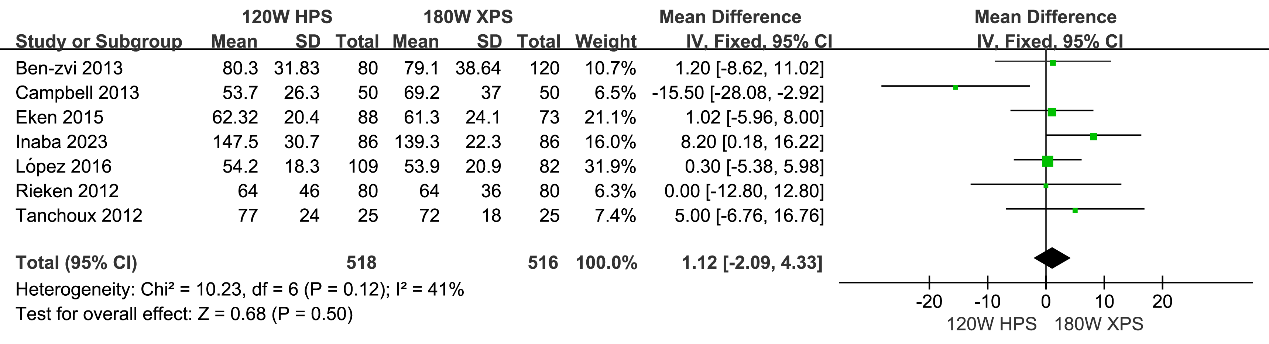


Fig.S1 Forest plot and meta-analysis of preoperative prostate volume.


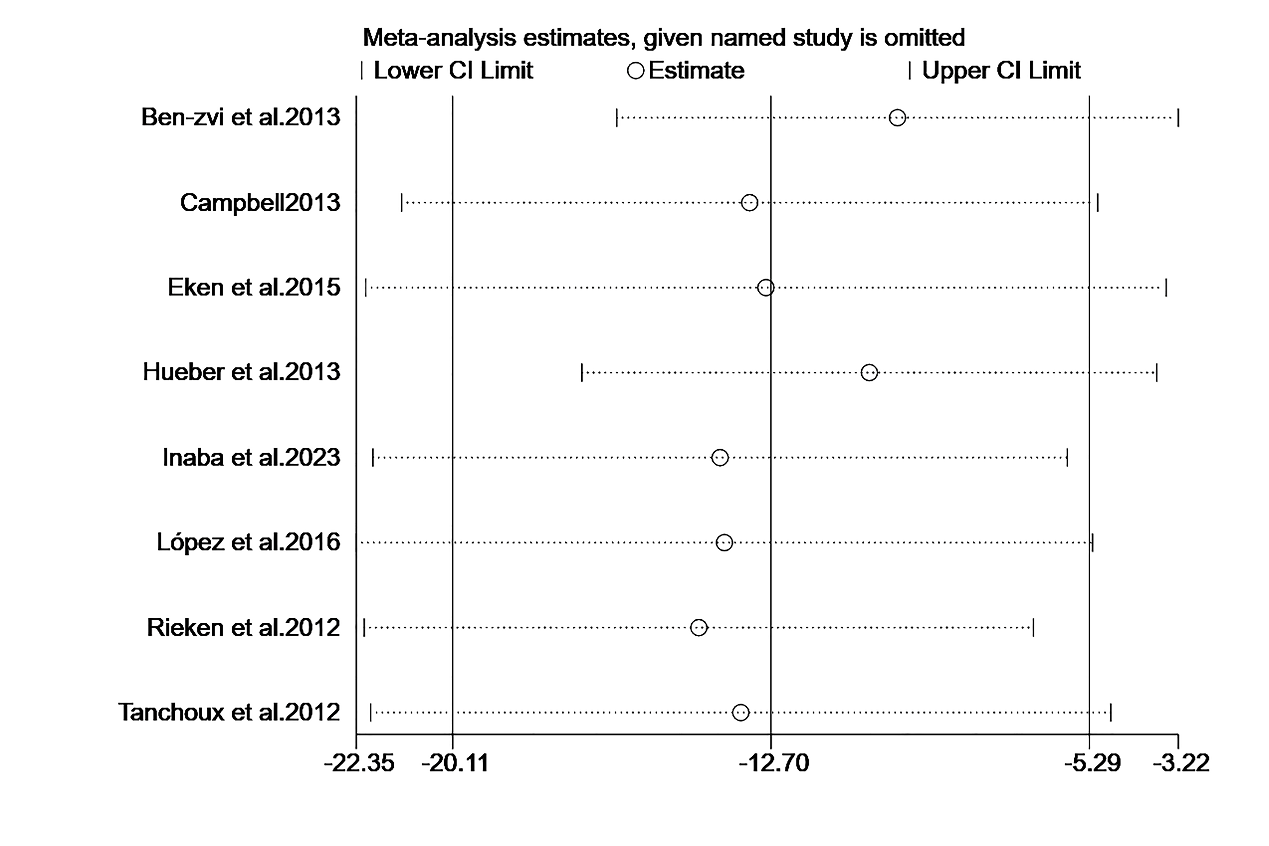
Fig.S2 Sensitivity analysis of operation time.


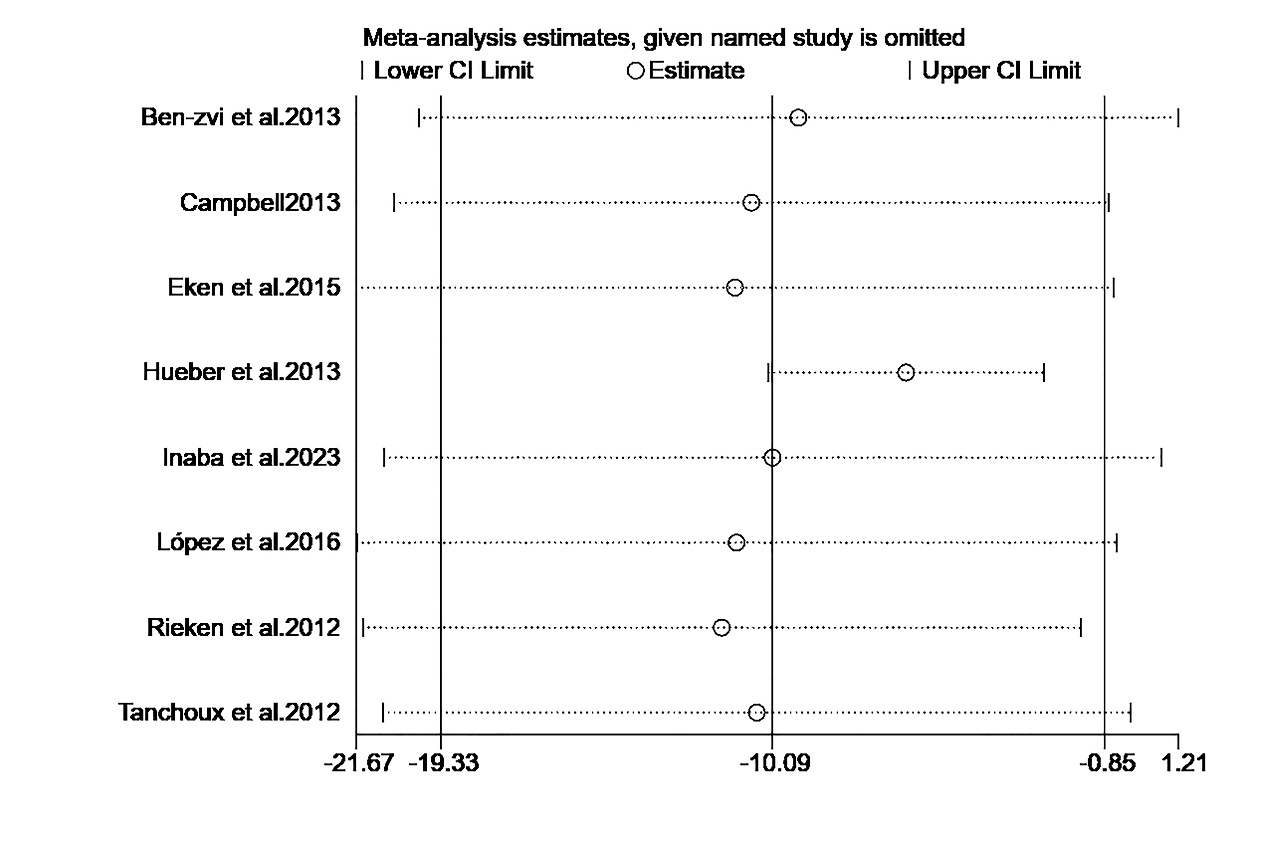
Fig.S3 Sensitivity analysis of lasing duration.


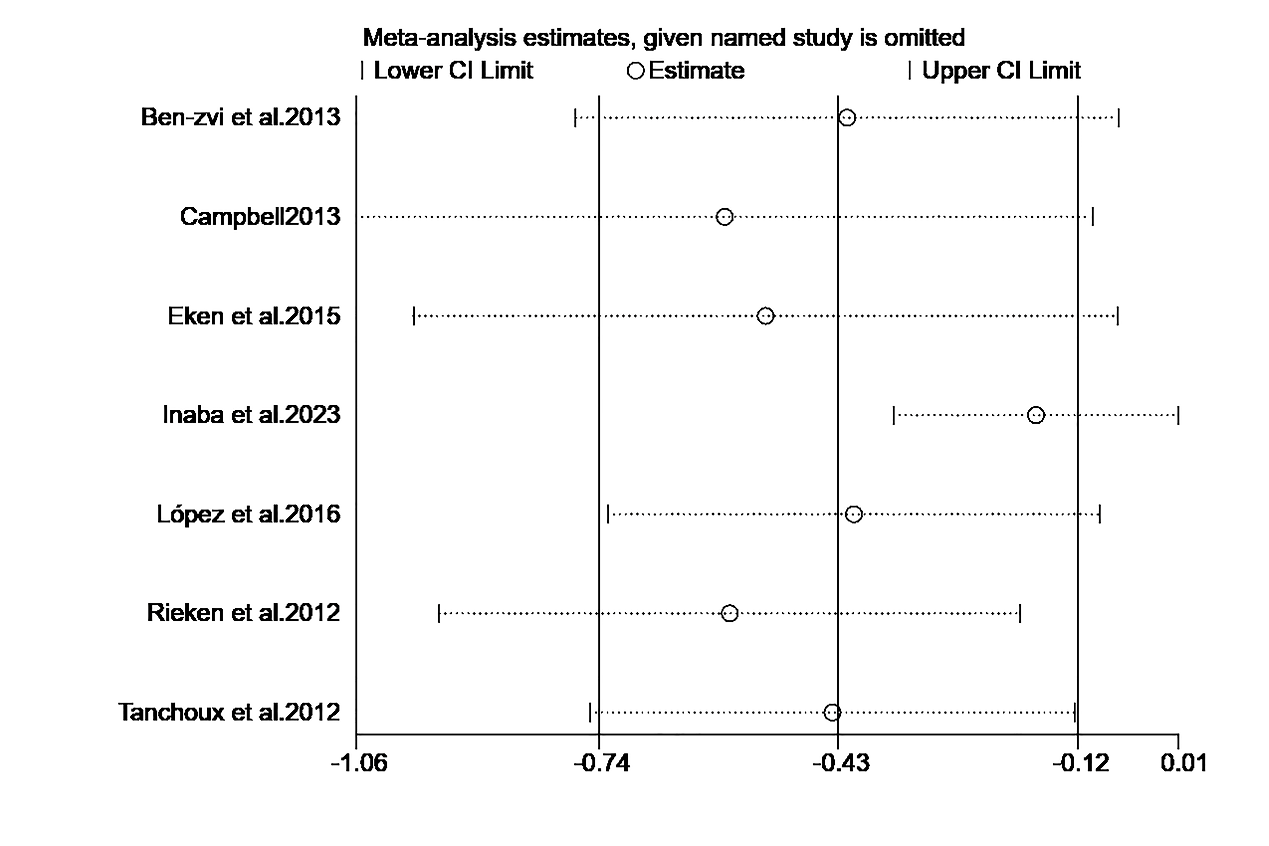
Fig.S4 Sensitivity analysis of postoperative catheterization duration.


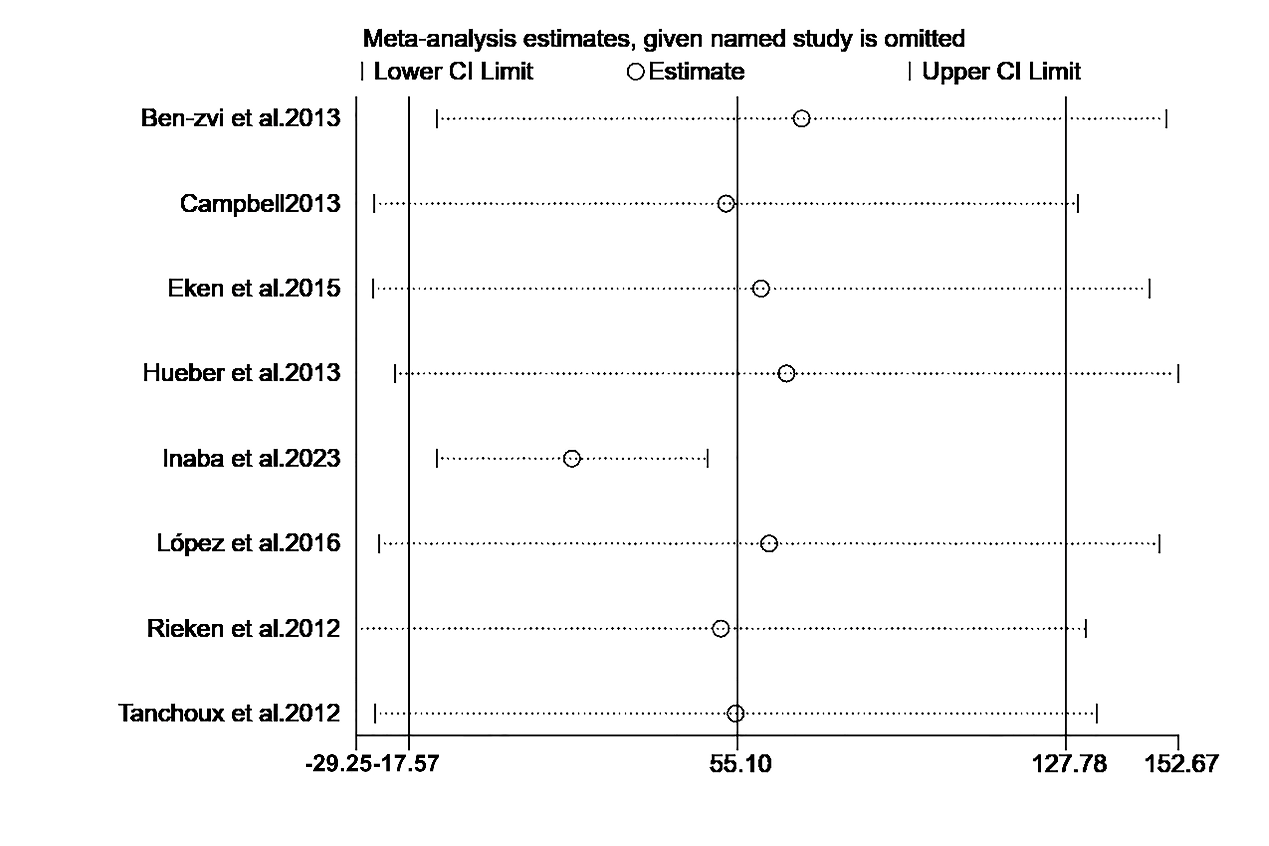
Fig.S5 Sensitivity analysis of energy consumption.


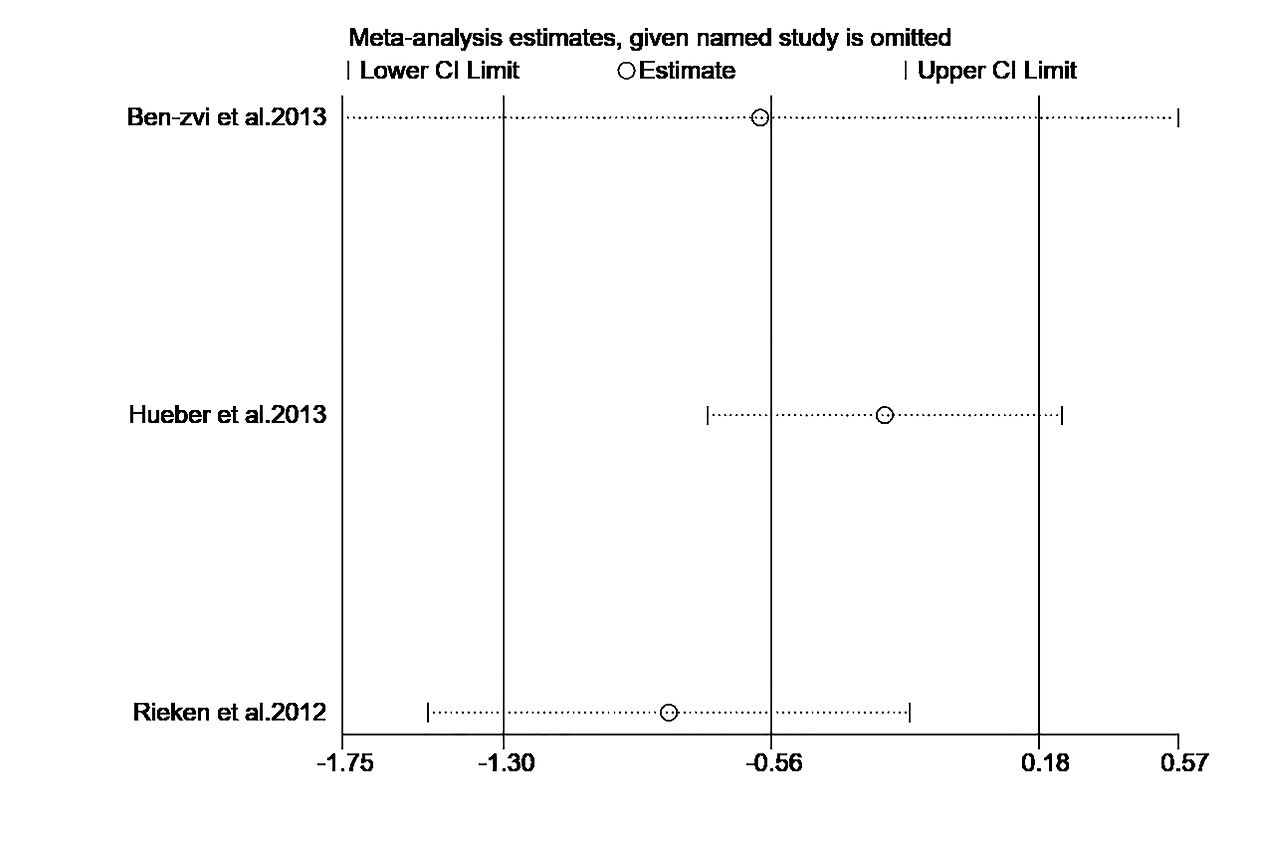
Fig.S6 Sensitivity analysis of laser fibers used.


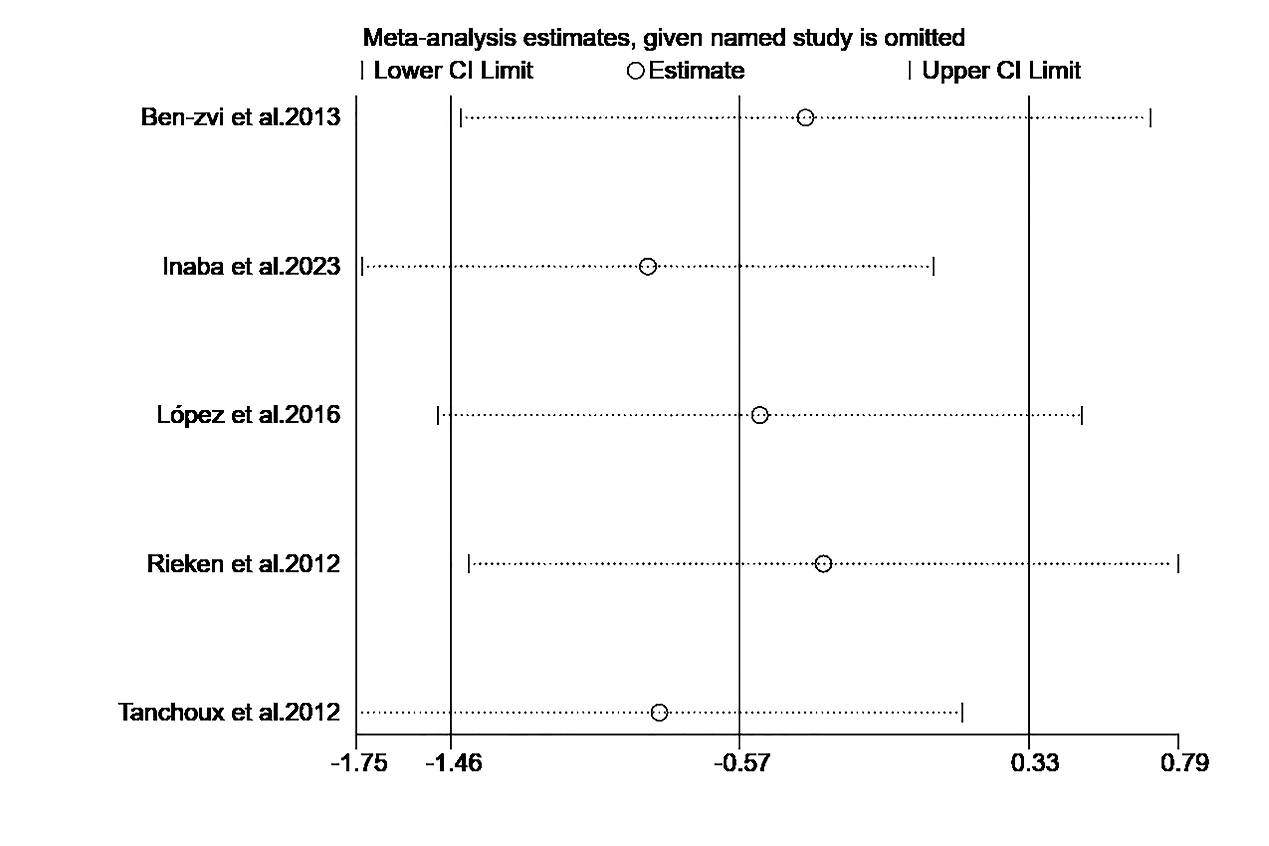
Fig.S7 Sensitivity analysis of LOS.


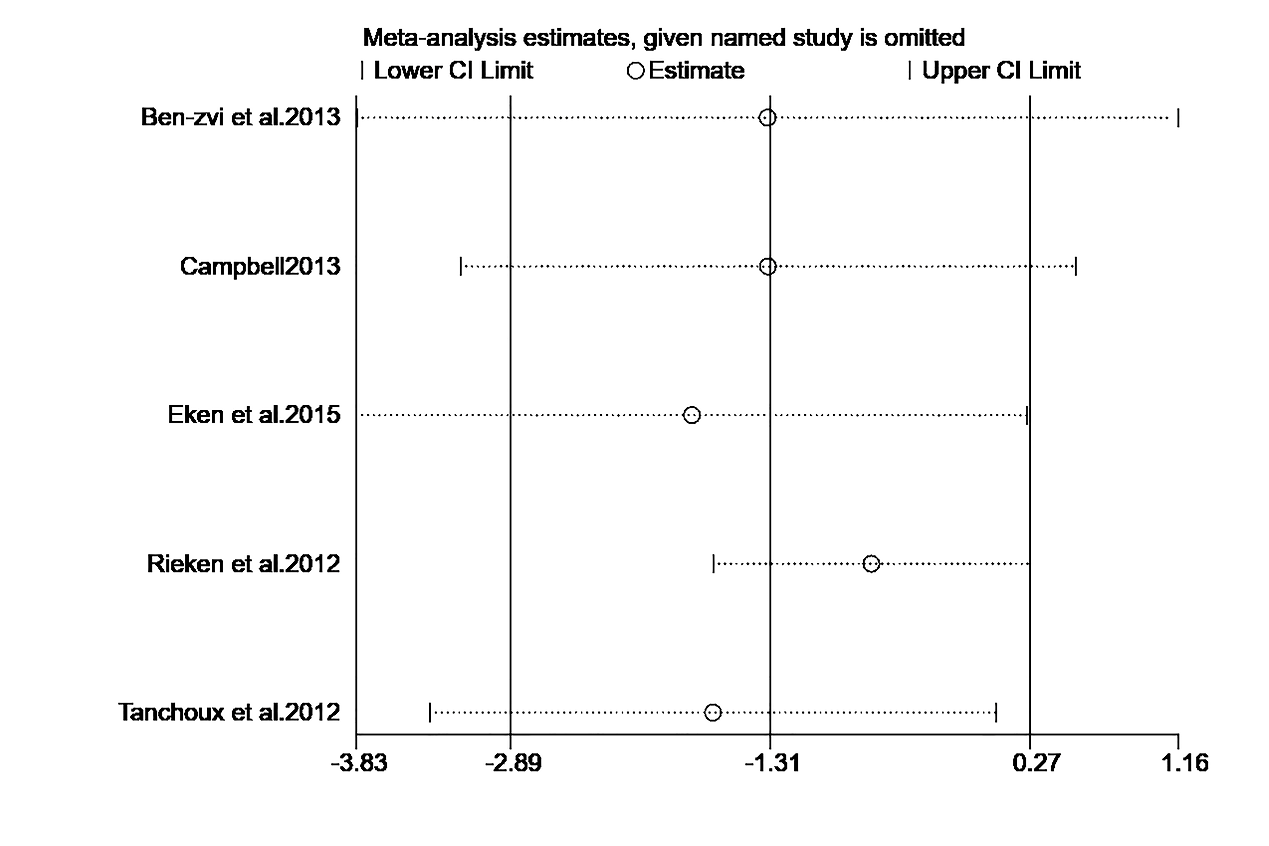
Fig.S8 Sensitivity analysis of IPSS improvement.


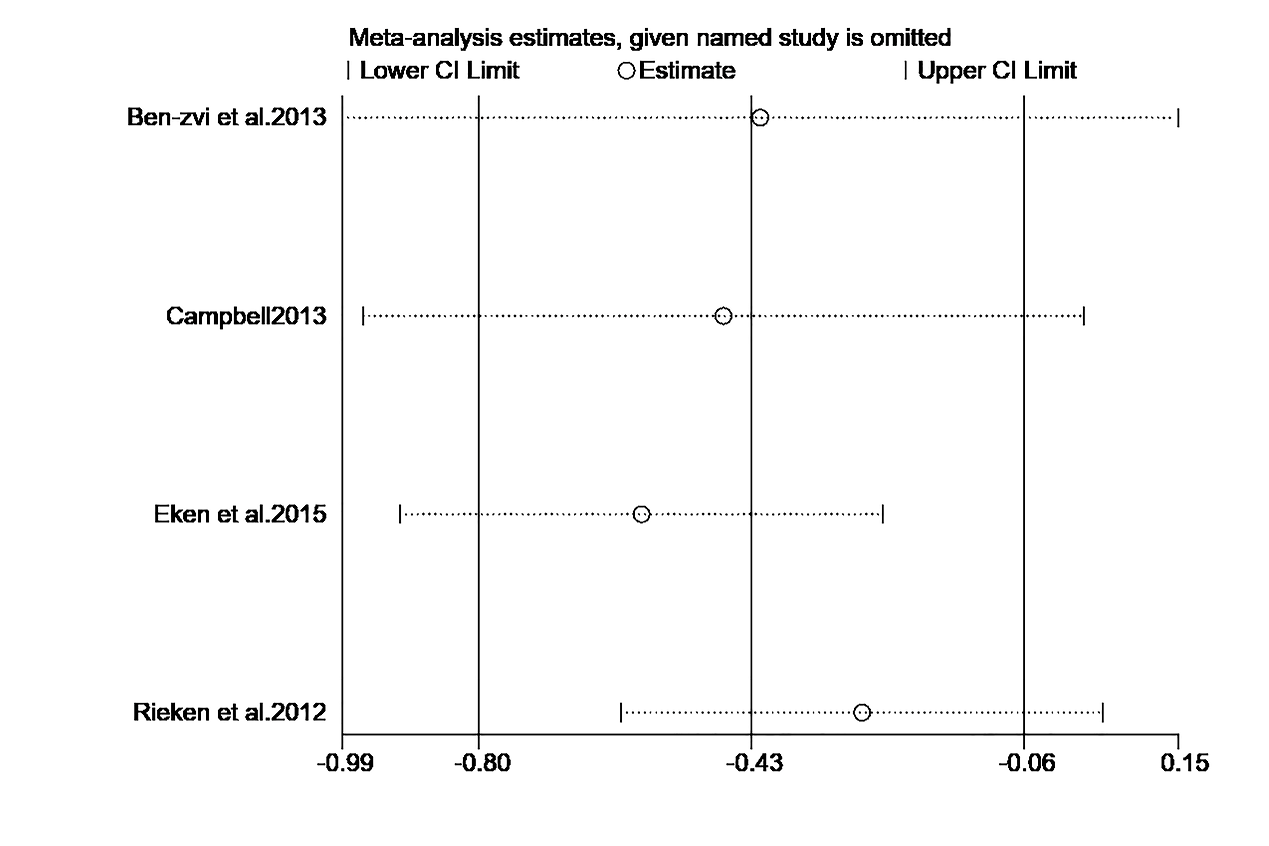


Fig.S9 Sensitivity analysis of QoL improvement.


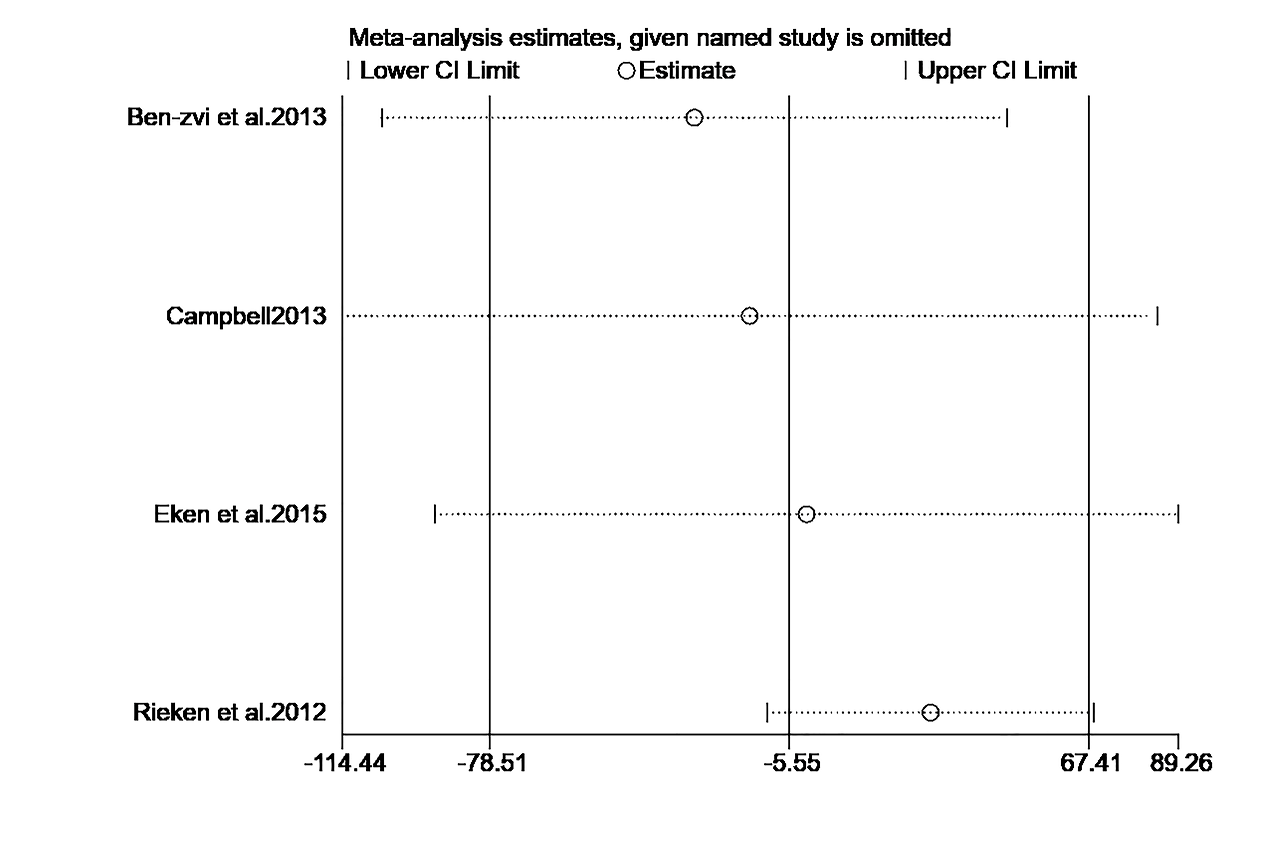
Fig.S10 Sensitivity analysis of PVR improvement.


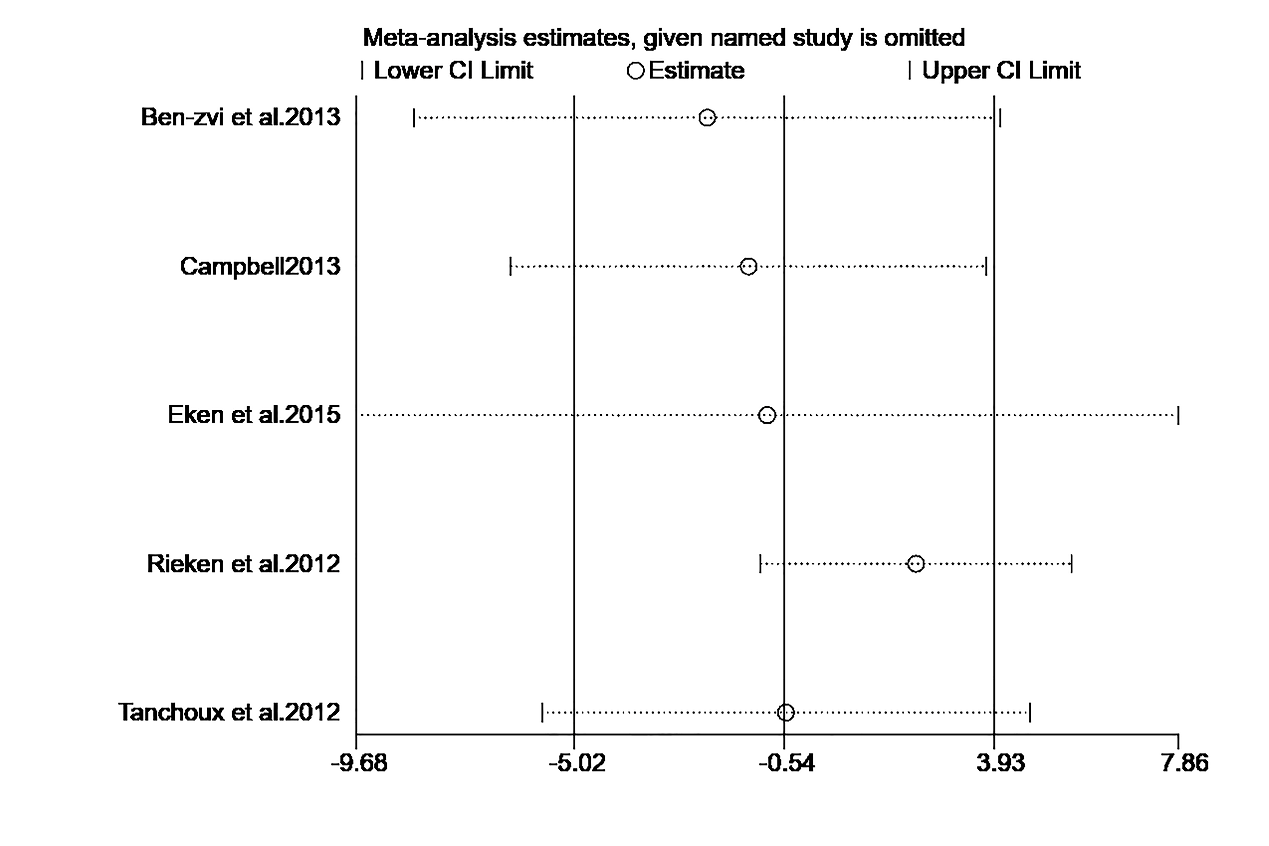


Fig.S11 Sensitivity analysis of Qmax improvement.
