# Supplementary material for: Comparative efficacy and safety of 180 W XPS vs. 120 W HPS GreenLight laser therapy for benign prostatic hyperplasia: a systematic review and meta-analysis
Source: PeerJ. 2024 Nov 27;12:e18615. doi: 10.7717/peerj.18615 (PMC11608016; doi:10.7717/peerj.18615)
Supplement: Supplemental Information 5 [file peerj-12-18615-s005.docx]

**English-Language Codebook**

| Chinese Term | English Translation |
| --- | --- |
| 变化 | Change |
| 尿道狭窄 | Urethral Stricture |
